# Supplementary material for: Through bonds or contacts? Mapping protein vibrational energy transfer using non-canonical amino acids
Source: Nat Commun. 2021 Jun 2;12:3284. doi: 10.1038/s41467-021-23591-1 (PMC8172543; doi:10.1038/s41467-021-23591-1)
Supplement: Supplementary file 1 — Supplementary Informations [file 41467_2021_23591_MOESM1_ESM.pdf]

Supplementary Informations

Through Bonds or Contacts? Mapping Protein Vibrational Energy Transfer Using Non-Canonical Amino Acids.

Erhan Deniz[1], Luis Valiño-Borau[2], Jan G. Löffler[1], Katharina B. Eberl[1], Adnan Gulzar[2], Steffen Wolf[2], Patrick M. Durkin[3,4], Robert Kaml[3], Nediljko Budisa[3,5], Gerhard Stock[2,*] and Jens Bredenbeck[1,*]

[1]Institute of Biophysics, Goethe University Frankfurt, 60438 Frankfurt/Main, Germany

[2]Biomolecular Dynamics, Institute of Physics, Albert Ludwigs University, 79104 Freiburg, Germany

[3]Institute of Chemistry, Technical University Berlin, Müller-Breslau-Straße 10, 10623 Berlin, Germany

[4]Present address: GenoSynth GmbH, Magnusstraße 11, 12489 Berlin

[5]Present address: Department of Chemistry, University of Manitoba, Winnipeg, R3T 2N2, Canada

*Corresponding authors: stock@physik.uni-freiburg.de, bredenbeck@biophysik.uni-frankfurt.de

CONTENT

SUPPLEMENTARY FIGURES................................2
SUPPLEMENTARY TABLES..................................6
SUPPLEMENTARY NOTES...................................7
SUPPLEMENTARY METHODS............................8
SUPPLEMENTARY REFERENCES........................11

SUPPLEMENTARY FIGURES

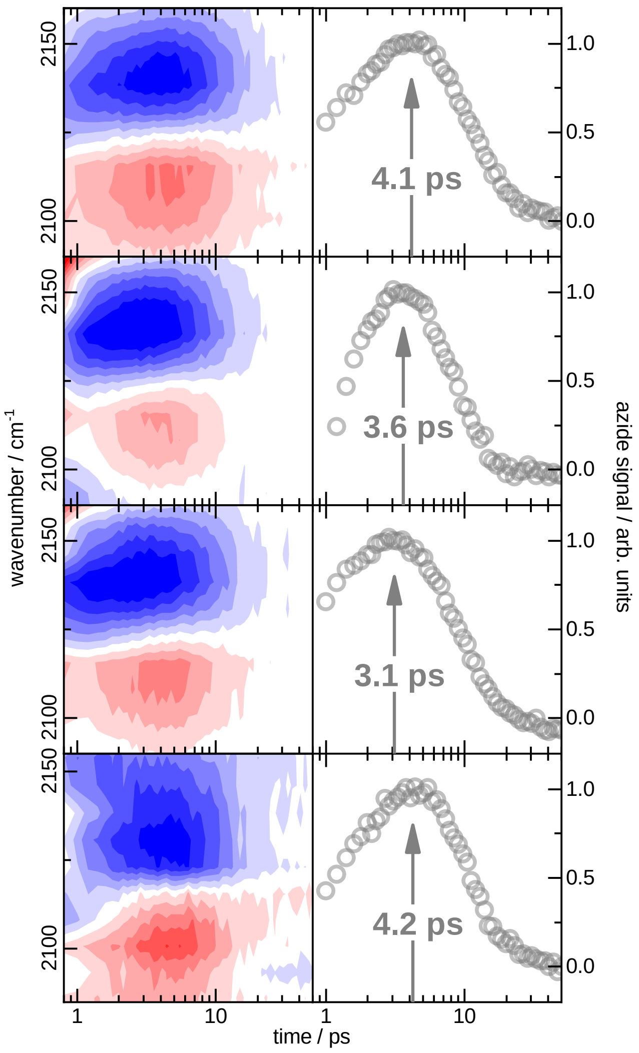

Supplementary Figure 1. TRIR spectra of TrpZip2 variants. Overview of TRIR spectra (left) and VET transients (right) of all four TrpZip2 variants, analogous to Figure 1b in the main text. Source data are provided as a Source Data file.

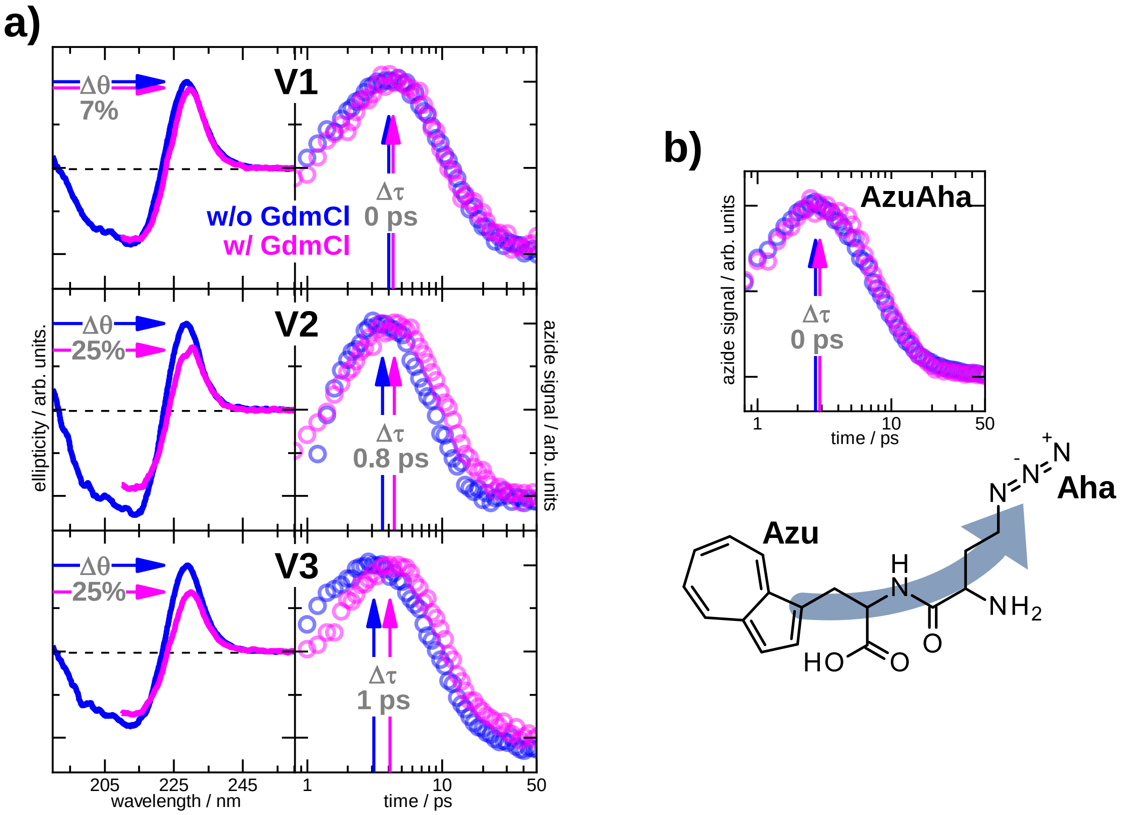

Supplementary Figure 2. Comparison of VET in the presence and absence of unfolding agent. a) Collection of CD spectra (left) and VET traces (right) of all across-strand TrpZip2 variants V1-V3. Without GdmCl (blue) and with 8 M GdmCl (magenta). On the left panels colored arrows point to the peak at ~ 228 nm, as a measure for the hairpin fold. Decrease in exciton signal intensity is noted in grey. On the right panels colored arrows point to the VET peak time. Delay of VET is noted in grey. a) VET trace (top) of the Azu-Aha dipeptide. In the structure formula (bottom) the blue arrow indicates the mainly contributing path for efficient VET, which is along the backbone via covalent bonds. For additional information on partial unfolding of TrpZip2 see according Supplementary Notes section below. Source data are provided as a Source Data file.

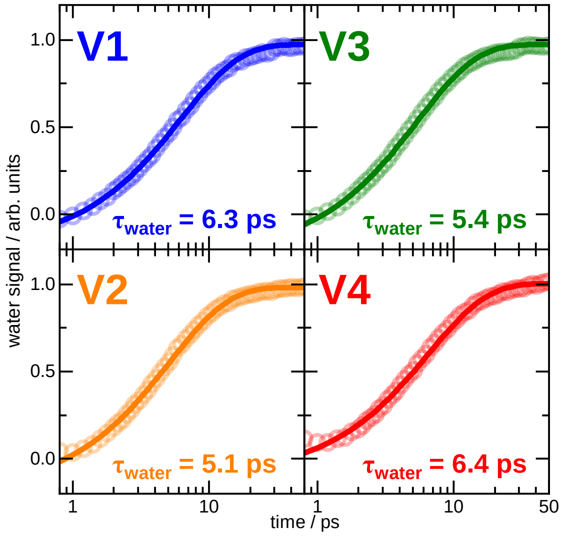

Supplementary Figure 3. VET transients of the water heating for all four TrpZip2 variants. The transients (colored circles) are fitted with a monoexponential function (colored solid line) to give us the experimentally determined rise times τ_{water}. For additional information on solvent dynamics see according Supplementary Notes section below. Source data are provided as a Source Data file.

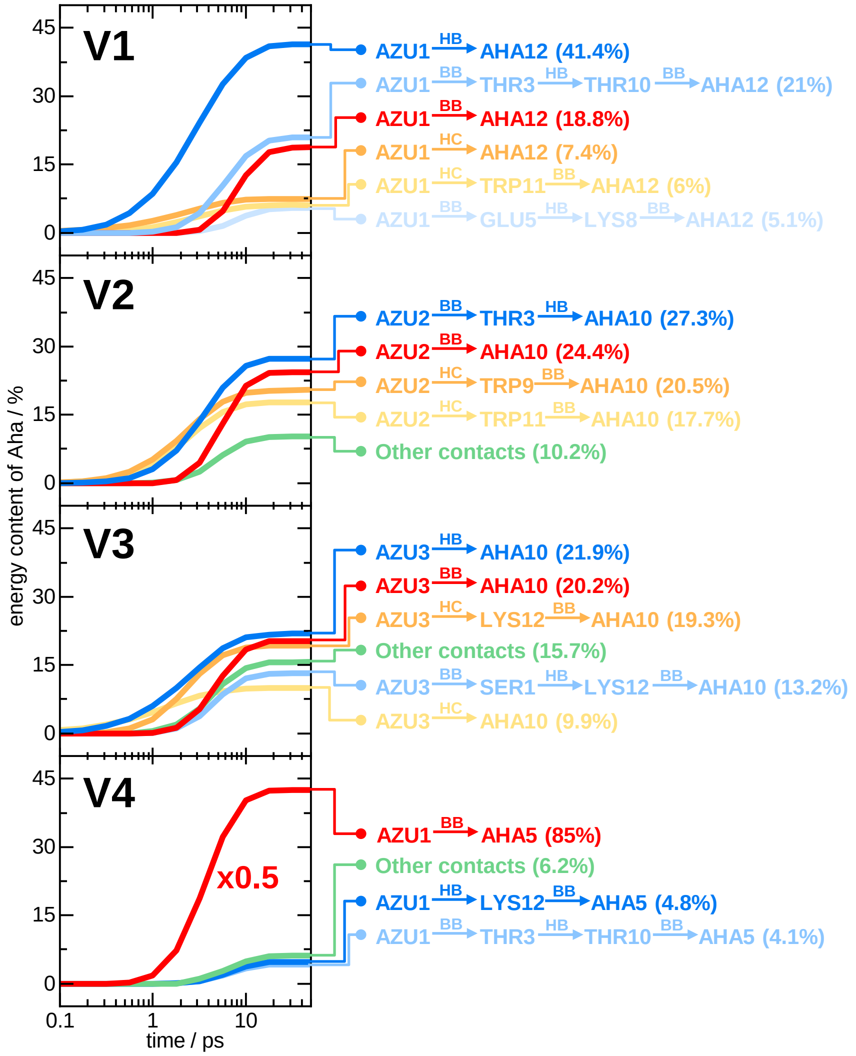

Supplementary Figure 4. Contribution of various pathways to the energy transport from the Azu heater to the Aha probe, obtained for the four variants of TrpZip2. Pathways may include the backbone (BB), interstrand hydrogen bonds (HB), and heater contacts (HC). For additional information on pathways see according Supplementary Notes section below. Source data are provided as a Source Data file.

SUPPLEMENTARY TABLES

Supplementary Table 1. List of systems, heater residues, and resulting parameters of the classical (left, red) and quantum-corrected (right, blue) master equation, including inverse heating rate $1/k_h$, solvent cooling time $1/k_{ps}$, solvent back-transfer time $1/k_{sp}$, and contact times $\tau_{ij} = 1/k_{ij}$ (all in units of ps). For additional information on master equation see according Supplementary Methods section below. Source data are provided as a Source Data file.

**Model**	**classical**				**quantum corrected**			
**System**	V1	V2	V3	V4	V1	V2	V3	V4
**Heater**	Azu1	Azu2	Azu3	Azu1	Azu1	Azu2	Azu3	Azu1
**$1/k_h$**	5.9	5.9	5.9	5.9	2.0	2.0	2.0	2.0
**$1/k_{ps}$**	7.5	8.3	8.3	8.9	6.3	5.1	5.4	6.4
**$1/k_{sp}$**	120	120	120	235	120	165	120	235
**Contact times**	$\tau_{1,12}$ = 9.1	$\tau_{1,12}$ = 110	$\tau_{1,12}$ = 7.7	$\tau_{1,12}$ = 11	$\tau_{1,12}$ = 3.0	$\tau_{1,12}$ = 37	$\tau_{1,12}$ = 2.6	$\tau_{1,12}$ = 3,7
	$\tau_{3,10}$ = 8.3	$\tau_{3,10}$ = 9.2	$\tau_{3,10}$ = 5.9	$\tau_{3,10}$ = 10	$\tau_{3,10}$ = 2.8	$\tau_{3,10}$ = 3.1	$\tau_{3,10}$ = 2.0	$\tau_{3,10}$ = 3,3
	$\tau_{5,8}$ = 9.1	$\tau_{5,8}$ = 9.1	$\tau_{5,8}$ = 9.1	$\tau_{5,8}$ = 9.1	$\tau_{5,8}$ = 3	$\tau_{5,8}$ = 3	$\tau_{5,8}$ = 3	$\tau_{5,8}$ = 3
	$\tau_{0,11}$ = 300	$\tau_{0,9}$ = 59	$\tau_{0,5}$ = 71	$\tau_{0,12}$ = 300	$\tau_{0,11}$ = 100	$\tau_{0,9}$ = 20	$\tau_{0,5}$ = 24	$\tau_{0,12}$ = 100
	$\tau_{0,12}$ = 300	$\tau_{0,11}$ = 77	$\tau_{0,10}$ = 125		$\tau_{0,12}$ = 100	$\tau_{0,11}$ = 26	$\tau_{0,10}$ = 42	
			$\tau_{0,12}$ = 43				$\tau_{0,12}$ = 14	

SUPPLEMENTARY NOTES

Partial unfolding of TrpZip2. If VET in a β-hairpin would only occur via the backbone, unfolding should not affect the peak times of those TrpZip2 variants, where the VET donor-sensor pair are attached at opposing strands, *i.e.* V1-V3. To test this hypothesis, we performed VET measurements on V1-V3 in presence of 8 M GdmCl—a strongly denaturing condition for the majority of peptides and proteins. The hydrophobic interactions of opposing tryptophans, however, renders the hairpin fold of TrpZip2 highly stable against chemical denaturation.

We managed to unfold V2 & V3 by only about 25% estimated from the exciton signal in the CD spectrum as shown in Supplementary Figure 2a. Nevertheless, the peak times are significantly shifted by about 1 ps indicating that across-strand VET must have occurred to a significant extend via contacts which get disturbed when high molarity of GdmCl is added. V1 remains almost unaffected by GdmCl and accordingly, a shift of VET peak time is not observed.

The VET transient of the Azu-Aha dipeptide is unchanged when 8 M GdmCl is added (see Supplementary Figure 2b). This confirms that the presence of GdmCl does not change the VET process, *e.g.* by changing energy transfer to the buffer.

Solvent dynamics. In our VET measurements, the energy which is injected into the system via the attached azulene moiety will at some point dissipate into the solvent, either directly from the azulene to the solvent or from other parts of the system, where the vibrational energy has been transferred to. Just as the azide moiety, the heated water gives rise to a VET signal occurring, however, on much longer timescales than the azide VET signal. This allows us to isolate the water contribution to determine its rise time τ_{water} (on average ~ 6ps) as shown in Supplementary Figure 3. Exact values are used for the rate model (see Supplementary Table 1 and Supplementary Methods).

Pathways. In the VET experiment we inject at time t = 0 vibrational energy into a specific residue i, and probe at a delayed time t the energy content $E_j(t)$ of some distant residue j. The energy flow from residue i to residue j may occur via several pathways, *e.g.* one pathway proceeding exclusively via the backbone and other pathways using a combination of backbone and contact transport. In practice, we are interested in the most efficient pathways that at a given time t have carried most of energy from i to j.

Since the rate matrix $\{k_{ij}\}$ of master equation Equation (2) completely accounts for the dynamics of the system, it also contains all information on energy transport pathways and their efficiency. Most straightforwardly, we obtain this information by running Markov chain Monte-Carlo simulations of the N-state (or N-residue) system. At each step, a random number is drawn which determines if the system remains in the current residue or changes to some other residue. In this way, we sample a stochastic trajectory in state space according to rate matrix $\{k_{ij}\}$. By counting how often the system has propagated from i to j along each possible pathway, the weights of these pathways are readily calculated. Supplementary Figure 4 shows the resulting pathways obtained from a quantum-corrected master equation for all four variants of VET donor and sensor position.

SUPPLEMENTARY METHODS

Sample synthesis. All peptides were synthesized on a Prelude Peptide Synthesizer by Gyros Protein Technologies (Uppsala, Sweden) using rink amide resin via standard Fmoc solid-phase peptide synthesis employing TBTU and Hünig's base as coupling reagents and DMF as solvent.

Rink Amide resin was purchased from Iris Biotech (Marktredwitz, Germany) with a potential loading of 0.45-0.75 mmol/gram. The resin was pre-swollen in DMF, then a solution of Fmoc-protected amino acid purchased from Iris Biotech (Marktredwitz, Germany) was dissolved in DMF and Hünigs base (4 eq. of each) and mixed for 30 min. The resin is then washed twice with DMF, once with CH_2Cl_2, then again with DMF and finally Fmoc-deprotection by treatment twice with a 20% piperidine solution in DMF for 15 min.

Canonical Fmoc-amino acids were double-coupled for 30 min each with 4 eq. of the amino acid and 4 eq. of TBTU and DIPEA in each coupling. Non-canonical Fmoc-amino acids were also double coupled, but for 40 min and with 1.2 eq. of Fmoc-amino acid and 4 eq. of DIPEA and HBTU. N-terminal deprotection of the growing peptide chain Fmoc group was achieved by treatment twice with a 20% piperidine solution in DMF for 15 min. All couplings and N-terminal deprotections were monitored using the standard Kaiser test methods and HPLC-MS of test-cleavages.

The peptides were sidechain-deprotected and cleaved from the resin under acidic conditions for 2 h v/v [93:4:1:1:1] CF_3COOH:H_2O:phenol/triisopropylsilane/2,20-(ethylenedioxy)-diethanethiol]. The peptides were then precipitated in ice-cold Et_2O, whereby the supernatant was then decanted, and the precipitate was dried under reduced pressure. Purification was achieved using reversed-phase HPLC on a 1260 Infinity system (Agilent Technologies) using a C18 preparative column (21.2 x 250 mm, 10 µm; Agilent Technologies) with gradients from 95% acetonitrile, 5% water, 0.1% CF_3COOH to 95% water, 5% acetonitrile, 0.1% CF_3COOH.

The peptides were characterized by electrospray ionization (ESI) mass spectrometry in positive ion mode on an LTQ Orbitrap XL mass spectrometer. A Grace Grom-Sil-120-ODS-4-HE (length, 50 mm; ID, 2 mm, 3 mm) column was used with a linear gradient of 20–100% over 10 min at 0.3 mL/min. The solvent system used was A (0.1% formic acid in H_2O) and B (0.1% formic acid in acetonitrile).

Sample preparation for CD spectroscopy. For CD spectra, lyophilized TrpZip2 variants were dissolved in in 20 mM phosphate buffer pH 2 to an optical density of ~ 0.8 at 228 nm, as measured by conventional UV/VIS absorption spectroscopy. For CD spectra in presence of GdmCl, the buffer was supplemented with 8 M GdmCl.

CD spectroscopy. CD spectra of the TrpZip2 variants without and with 8 M GdmCl were recorded with a Jasco J720 spectrometer in thin-layer CaF_2 cells with an optical path length of 5 µm. The CD spectra were normalized to the UV/VIS absorption at 228 nm, which was recorded with a Hitachi U-2000 spectrophotometer in the same thin-layer cell.

Sample preparation for laser measurements. Lyophilized TrpZip2 variants were dissolved in 20 mM phosphate buffer pH 2 to a concentration of approximately 15 mg/ml. For measurements in presence of GdmCl the buffer was prepared with 8 M GdmCl. The peptide solution was centrifuged to get rid of any precipitant. Afterwards the sample was loaded into a custom-built mountable CaF_2 cell with 100 µm path length.

Laser measurements. A Ti:Sa regenerative amplifier system (Millennia, Tsunami, Empower, Spitfire) from Spectra Physics with 1 kHz repetition rate generating 800 nm pulses with 100 fs pulse duration and 3 mJ pulse energy was used to pump two home-built optical parametric amplifiers (OPAs). With one OPA we generated our mid-IR probe and reference pulses via difference frequency generation. Both pulses were guided through the sample, but hit the sample at spatially separated positions. Both pulses were dispersed on a spectrometer (Triax, Jobin Yvon) with a grating of 150 lines per millimeter and detected on a 2 x 32 pixels MCT detector (Infrared Associates) cooled with an automated liquid nitrogen refilling system.[1] The detector signal was amplified and integrated with home-built electronics.

After a second OPA we generated our 613 nm pump pulse via second harmonic generation in a β-barium-borate (BBO) crystal. A mechanical chopper blocked every other pulse resulting in pump-on/pump-off difference spectra. The polarization angle between the pump and probe pulse was set to the magic angle with a half-wave plate and a polarizer to get rid of contributions from rotational diffusion. The pump and probe pulse diameters were 150 µm and 65 µm FWHM, respectively. The pump pulse energy was set to 8 µJ. The sample was loaded into a rotating and vertically moving cell with two CaF_2 windows and a 100 µm spacer in-between.

Data acquisition and analysis. Transient IR spectra were recorded with software written in Visual Basic 6 and LabVIEW 2016. Data was analyzed with MatLab R2018a (MathWorks) and OriginPro 2018 (OrginLab). The spectrum at -20 ps delay time was subtracted from every other time point of the TRIR spectrum as a background. In addition to that constant contribution, the azide signal is overlapping with a broad time dependent water signal caused by solvent heating. To subtract the water signal, we averaged the spectra after 54 ps delay time, where the azide signal has decayed, to a representative water spectrum. Note, that the signals at that late delay times is only due to solvent heating. This averaged water spectrum was fitted to the spectrum at every delay time by adjusting the amplitude and offset and then subtracted such that we are left with the azide signal. We calculated the total VET-induced azide absorption change by adding up the absolute absorption change at all pixels carrying the azide VET signal.

MD simulations. Gulzar *et al.*[2] recently presented extensive non-equilibrium MD simulations of the energy flow in TrpZip2[3] (PDB entry 1LE1). All MD simulations were performed using GROMACS[4] package v2016.3, Amber99sb*ILDN forcefield[5–7] and TIP3P water.[8] Parameters for Azu and Aha were reported in Supplementary Ref. 2 and 9, respectively. Na^+ and Cl^- were added at a salt concentration of 0.1 M, with an excess of Cl^- to compensate the net positive charge (+2) of TrpZip2. Long-range electrostatic interactions (distances > 1.2 nm) were computed by the Particle Mesh Ewald (PME) method,[10] short-range electrostatic interactions were treated explicitly using a Verlet cut-off scheme. After energy minimization, a 10 ns NPT equilibration run was performed. In all equilibrium simulations, we used an integration time step of 2 fs and maintained a temperature of 300 K (via the Bussi thermostat,[11] coupling time 0.1 ps) and a pressure of 1 bar (via the Berendsen barostat,[12] coupling time 0.1 ps). Following suitable equilibrium runs for each TrpZip2 variant considered at T_0 = 300 K (100 ns length each), N_{traj} = 5000 statistically independent initial structures were stored for the subsequent non-equilibrium runs. To mimic the initial heating of azulene via electronic excitation and subsequent ultrafast (~1 ps) internal conversion[13], the resulting vibrational excitation was approximated by an instantaneous temperature jump, where the excess energy $k_B \Delta T$ is chosen to match the $S_0 \rightarrow S_1$ excitation energy of ~2 eV, resulting in $\Delta T \approx 600$ K. Following the heating of Azu to $T_0+\Delta T$, non-equilibrium MD simulations of 50 – 100 ps length were performed. It was found that *NVT* simulations (time step δt = 0.7 fs) with only the solvent coupled to the thermostat (coupling constant τ_T = 10 ps) represents an efficient and accurate strategy.[2]

To monitor the flow of vibrational energy from the heater residue through the protein, we consider the time evolution of the kinetic energy of the *i*-th residue, $E_i^{kin} = \sum_j E_{i,j}^{kin}(t)$, where the sum runs over all atoms *j* of residue *i*. The time-dependent expectation value of the kinetic energy per degree of freedom, $E_i(t)$, is calculated via an ensemble average over N_{traj} non-equilibrium trajectories,

$$E_i(t) = \frac{1}{f_i N_{traj}} \sum_{n=1}^{N_{traj}} E_i^{kin}(n,t) - E_i^{eq}, \qquad (1)$$

where f_i denotes the degrees of freedom of residue *i*. Since $E_i^{eq} = k_B T/2$ is the equilibrium energy per degree of freedom, $E_i(t)$ is expected to decay to zero at long times.

Master equation model. To describe the flow of vibrational energy in proteins, Buchenberg *et al.*[14] suggested a master equation

$$\frac{dE_j(t)}{dt} = \sum_i \left[k_{ij} E_i(t) - k_{ji} E_j(t)\right], \qquad (2)$$

where E_i denotes the kinetic energy of residue *i* and k_{ij} represents the rate of energy transport from residue *i* to residue *j*. The model is valid in the case of diffusive energy transport,[14] which is typically found for solvated biomolecules.[15]

Due to the large number of transport rates (~ N^2 with *N* being the number of protein residues), a direct fit of the master equation to MD results is ill-defined and likely to yield non-physical results. On this account, Buchenberg *et al.*[14] derived scaling rules, which aim to describe energy transport rates in terms of a few parameters. In particular, by exploiting the equivalence of the master equation and a discrete diffusion equation, a scaling rule for the energy transport between two adjacent backbone residues *i* and *j* = *i*-1 was derived,

$$k_{ij} = \frac{D_B}{\langle x_{ij}^2 \rangle} \sqrt{\frac{f_j}{f_i}}, \qquad (3)$$

where D_B denotes the backbone diffusion coefficient and f_i denotes the degrees of freedom of residue *i*. $\langle x_{ij}^2 \rangle$ represents the average square distance between every pair of atoms of residues *i* and *j* along covalent bonds, which reflects the average distance energy has to travel among every atom of both residues. The factor $\sqrt{f_j/f_i}$ assures that the rates obey the detailed

balance relation $k_{ij} f_i = k_{ji} f_j$. Employing distances commonly found for peptides and D_B = 1.1 nm²ps⁻¹, typical transfer times between adjacent residues are $1/k_{ij}$ = 0.5 – 1 ps.[16]

Moreover, a scaling rule for the energy transport via polar contacts was derived,[16] using a multidimensional model that describes backbone transport and contact transport as diffusion processes in different directions using different energy diffusion constants. In direct analogy to scaling rule (3) for backbone transport, we then obtain for the contact transport rate

$$k_{ij}^C = \frac{D_C}{\langle q_{ij}^2 \rangle} \sqrt{\frac{f_j}{f_i}}, \qquad (4)$$

where D_C denotes the contact diffusion constant and $\langle q_{ij}^2 \rangle$ represents the mean square distance of the two contact atoms. Typical contact transfer times are 0.6 – 30 ps.[16] The contact, solvent and intra-residue rates used for all four variants of TrpZip2 in the classical model are summarized in Supplementary Table 1. Backbone transport was found to obey a diffusive scaling rule with a diffusion constant D_B = 1.1 nm^2ps^{-1}. All 1-12, 3-10 and 5-8 contacts are β-sheet-stabilizing hydrogen bonds, the remaining contacts being heater contacts (signaled by the index of the heater '0'). In most cases, these heater contacts are of van der Waals/hydrophobic or stacking nature; however, the 0-12 heater contact in V3 is a cation-aryl contact (the charged tip of Lys12 and the adjacent ε group are attracted by the negatively charged ring center of Azu), and the 3-10 contact in V3 is a dipole-dipole contact. Both heater contacts in V2 are stacking contacts with Trp rings, while the remaining contacts in V1 and V4 are of van der Waals/hydrophobic nature in their dominant conformations. From the comparison of the hydrogen bond rates to several geometrical properties of these contacts, a scaling rule was found relating the inverse mean square distance to the rate value, with a diffusion constant D_C = $2.1 \cdot 10^{-3}$ nm^2ps^{-1}. As discussed in detail by Valiño-Borau *et al.*,[16] the resulting master equation results for all four variants of TrpZip2 are in excellent agreement with the corresponding non-equilibrium MD simulations.

Quantum corrections. Based on a comparison of classical and quantum-mechanical perturbation theory, the validity of classical non-equilibrium molecular dynamics simulations to describe vibrational energy redistribution in biomolecules was studied in our previous work.[17] Adopting well-established models of vibrational relaxation, explicit expressions of quantum correction factors were derived, that are to be applied to the results of classical simulations in order to match the correct quantum results.

In this work, an empirical quantum correction was determined by fitting master equation results to experimental peak times. To this end, we applied the quantum correction factor Q to the coefficients D_B and D_C for backbone and contact transport as well as the heating rate k_h and heater contact rates and performed a global fit of Q such that the quantum-corrected master equation results reproduces best the experimental peak times. In this way we obtained a quantum correction factor of 3.1. Details of the fitting procedure are described in Supplementary Ref. 18. Since the water dissipation times $1/k_{ps}$ of all systems were found to be quite similar in experiment (~ 6ps) and in MD (~ 8ps), we took the experimental values for the final model. The resulting parameters of the quantum-corrected master equation are comprised in Supplementary Table 1.

SUPPLEMENTARY REFERENCES

1. Deniz, E., Eberl, K. B. & Bredenbeck, J. Note: An automatic liquid nitrogen refilling system for small (detector) Dewar vessels. *Review of Scientific Instruments* **89,** 116101 (2018).
2. Gulzar, A., Valiño Borau, L., Buchenberg, S., Wolf, S. & Stock, G. Energy transport pathways in proteins: A nonequilibrium molecular dynamics simulation study. *J. Chem. Theory Comput.,* 5750–5757 (2019).
3. Cochran, A. G., Skelton, N. J. & Starovasnik, M. A. Tryptophan zippers: Stable, monomeric beta-hairpins. *Proc. Natl. Acad. Sci. U.S.A.* **98,** 5578–5583 (2001).
4. Abraham, M. J. *et al.* GROMACS: High performance molecular simulations through multi-level parallelism from laptops to supercomputers. *SoftwareX* **1,** 19–25 (2015).
5. Hornak, V. *et al.* Comparison of multiple Amber force fields and development of improved protein backbone parameters. *Proteins* **65,** 712–725 (2006).
6. Best, R. B. & Hummer, G. Optimized molecular dynamics force fields applied to the helix-coil transition of polypeptides. *J. Phys. Chem. B* **113,** 9004–9015 (2009).
7. Lindorff-Larsen, K. *et al.* Improved side-chain torsion potentials for the Amber ff99SB protein force field. *Proteins* **78,** 1950–1958 (2010).
8. Jorgensen, W. L., Chandrasekhar, J., Madura, J. D., Impey, R. W. & Klein, M. L. Comparison of simple potential functions for simulating liquid water. *J. Chem. Phys.* **79,** 926–935 (1983).
9. Stucki-Buchli, B. *et al.* 2D-IR Spectroscopy of an AHA Labeled Photoswitchable PDZ2 Domain. *J. Phys. Chem. A* **121,** 9435–9445 (2017).
10. Darden, T., York, D. & Pedersen, L. Particle mesh Ewald: An N·log(N) method for Ewald sums in large systems. *J. Chem. Phys.* **98,** 10089–10092 (1993).
11. Bussi, G., Donadio, D. & Parrinello, M. Canonical sampling through velocity rescaling. *J. Chem. Phys.* **126,** 14101 (2007).
12. Berendsen, H. J. C., Postma, J. P. M., van Gunsteren, W. F., DiNola, A. & Haak, J. R. Molecular dynamics with coupling to an external bath. *J. Chem. Phys.* **81,** 3684–3690 (1984).
13. A. J. Wurzer, T. Wilhelm, J. Piel & E. Riedle. Comprehensive measurement of the S1 azulene relaxation dynamics and observation of vibrational wavepacket motion. *Chem. Phys. Lett.* **299,** 296–302 (1999).
14. Buchenberg, S., Leitner, D. M. & Stock, G. Scaling Rules for Vibrational Energy Transport in Globular Proteins. *J. Phys. Chem. Lett.* **7,** 25–30 (2016).
15. Botan, V. *et al.* Energy transport in peptide helices. *Proc. Natl. Acad. Sci. U.S.A.* **104,** 12749–12754 (2007).
16. Valiño Borau, L., Gulzar, A. & Stock, G. Master equation model to predict energy transport pathways in proteins. *J. Chem. Phys.* **152,** 45103 (2020).
17. Stock, G. Classical simulation of quantum energy flow in biomolecules. *Phys. Rev. Lett.,* 118301 (2009).
18. Luis Valiño Borau. PhD Thesis. University of Freiburg (2020). https://freidok.uni-freiburg.de/data/194054
