# Supplementary material for: Through bonds or contacts? Mapping protein vibrational energy transfer using non-canonical amino acids
Source: Nat Commun. 2021 Jun 2;12:3284. doi: 10.1038/s41467-021-23591-1 (PMC8172543; doi:10.1038/s41467-021-23591-1)
Supplement: Supplementary file 3 — Reporting Summary [file 41467_2021_23591_MOESM3_ESM.pdf]

nature research

Corresponding author(s): Gerhard Stock, Jens Bredenbeck

Last updated by author(s): Apr 27, 2021

Reporting Summary

Nature Research wishes to improve the reproducibility of the work that we publish. This form provides structure for consistency and transparency in reporting. For further information on Nature Research policies, see our Editorial Policies and the Editorial Policy Checklist.

Statistics

For all statistical analyses, confirm that the following items are present in the figure legend, table legend, main text, or Methods section.

n/a	Confirmed	
[x]	[ ]	The exact sample size (n) for each experimental group/condition, given as a discrete number and unit of measurement
[x]	[ ]	A statement on whether measurements were taken from distinct samples or whether the same sample was measured repeatedly
[x]	[ ]	The statistical test(s) used AND whether they are one- or two-sided<br>*Only common tests should be described solely by name; describe more complex techniques in the Methods section.*
[x]	[ ]	A description of all covariates tested
[x]	[ ]	A description of any assumptions or corrections, such as tests of normality and adjustment for multiple comparisons
[x]	[ ]	A full description of the statistical parameters including central tendency (e.g. means) or other basic estimates (e.g. regression coefficient) AND variation (e.g. standard deviation) or associated estimates of uncertainty (e.g. confidence intervals)
[x]	[ ]	For null hypothesis testing, the test statistic (e.g. F, t, r) with confidence intervals, effect sizes, degrees of freedom and P value noted<br>*Give P values as exact values whenever suitable.*
[x]	[ ]	For Bayesian analysis, information on the choice of priors and Markov chain Monte Carlo settings
[x]	[ ]	For hierarchical and complex designs, identification of the appropriate level for tests and full reporting of outcomes
[x]	[ ]	Estimates of effect sizes (e.g. Cohen's d, Pearson's r), indicating how they were calculated

Our web collection on statistics for biologists contains articles on many of the points above.

Software and code

Policy information about availability of computer code

Data collection	Experimental data on our femtosecond pump-probe measurements were collected with custom-made software written in Visual Basic 6 and LabView 2016. MD simulations were carried out with Gromacs v2016.3.
Data analysis	Experimental data on our laser measurements were analyzed with MatLabR2018a from MathWorks and OriginPro2018 from OriginLab. MD trajectories were analysed with Gromacs v2016.3. Master equation model building and MCMC calculation was performed as outlined in Ref. [Valino2020].

For manuscripts utilizing custom algorithms or software that are central to the research but not yet described in published literature, software must be made available to editors and reviewers. We strongly encourage code deposition in a community repository (e.g. GitHub). See the Nature Research guidelines for submitting code & software for further information.

Data

Policy information about availability of data

All manuscripts must include a data availability statement. This statement should provide the following information, where applicable:

- Accession codes, unique identifiers, or web links for publicly available datasets
- A list of figures that have associated raw data
- A description of any restrictions on data availability

The data sets collected and analyzed for the reported experiments and simulations are available from the corresponding authors upon reasonable request. A reporting summary for this article is available. Source data are provided with this paper. Atomic coordinates of the original TrpZip2 peptide the simulations are based on can be found in the Protein Data Bank under the accession code PDB 1IL1 (Ref. 23).

Field-specific reporting

Please select the one below that is the best fit for your research. If you are not sure, read the appropriate sections before making your selection.

☒ Life sciences ☐ Behavioural & social sciences ☐ Ecological, evolutionary & environmental sciences

For a reference copy of the document with all sections, see nature.com/documents/nr-reporting-summary-flat.pdf

Life sciences study design

All studies must disclose on these points even when the disclosure is negative.

Sample size	Femtosecond pump-probe experiments: for each of the 4 measured TrpZip2 variants, ~300 000 laser shots were averaged per transient spectrum at each time delay. For the Azu-Aha dipeptide ~50 000 laser shots were averaged per transient spectrum at each time delay. These sample sizes where chosen to obtain smooth transients and very good signal to noise (minimum of 20/1) so that the time of maximal signal could be clearly determined.<br>MD simulations: for each of the 4 simulated TrpZip2 variants, 5000 statistically independent simulations of 50 ps length were carried out. This sample size was sufficient so that time-dependent means of residue energies exhibited clearly distinguishable maxima with very good signal to noise (minimum of 2/1).
Data exclusions	No datasets were excluded from analysis.
Replication	Femtosecond pump-probe experiments: Azu-Aha VET transients were measured several times over the course of weeks, using ~100 scans through all delay times, succesfully showing that VET times are reproducible within 0.2 ps with our setup. VET transients of all TrpZip2 variants are the result of >400 scans through all delay times, aquired over several hours on the same sample, respectively.<br>MD simulations: All of the 5000 numerical simulations represent individual statistically independent computational experiments, which were run on different computation cluster nodes at different times over several weeks and therefore explicitly include replication.
Randomization	Femtosecond pump-probe experiments: Randomization was not performed, because our study used a set of four proteins, separate measurements were carried out and analyzed for each protein and no grouping of protein samples or data was done.<br>MD simulations: Randomization was not applicable in evaluation of MD data, as no separation of data into groups was performed, nor is there a possible source of bias of MD simulation data by the researcher setting up and evaluating these simulations.
Blinding	Femtosecond pump-probe experiments: Blinding was not performed, because the experimental data where recorded using objective instruments and there was no possible source of bias by the researcher carrying out the measurements and the data evaluation.<br>MD simulations: Blinding was not applicable in evaluation of MD data, as neither a separation of data into groups was performed, nor is there a possible source of bias of MD simulation data by the researcher setting up and evaluating these simulations.

Reporting for specific materials, systems and methods

We require information from authors about some types of materials, experimental systems and methods used in many studies. Here, indicate whether each material, system or method listed is relevant to your study. If you are not sure if a list item applies to your research, read the appropriate section before selecting a response.

Materials & experimental systems

n/a	Involved in the study	
☒	☐	Antibodies
☒	☐	Eukaryotic cell lines
☒	☐	Palaeontology and archaeology
☒	☐	Animals and other organisms
☒	☐	Human research participants
☒	☐	Clinical data
☒	☐	Dual use research of concern

Methods

n/a	Involved in the study	
☒	☐	ChIP-seq
☒	☐	Flow cytometry
☒	☐	MRI-based neuroimaging
